# SARS-CoV-2 at the human-pet interface: transmission and pre-existing immunity in pets within infected households

**DOI:** 10.3389/fvets.2026.1862845

**Published:** 2026-07-20

**Authors:** Beatriz Davinia Tomeo-Martín, Ana Mendez-Echevarria, Pablo Delgado-Bonet, Miguel Ángel Jiménez-Clavero, Ana de la Torre, Cristina Calvo, Elisa Pérez-Ramírez, Jovita Fernández-Pinero, Francisco Llorente, Talía Sainz, Pilar Aguilera-Sepúlveda, Sonia Alcolea, Lucía Escolano, Cristina Cano-Gómez, Irene Iglesias, Ana Judith Perisé-Barrios

**Affiliations:** 1 Unidad de Investigación Biomédica (UIB-UAX), Universidad Alfonso X el Sabio, Madrid, Spain; 2 Department of Pediatric Infectious and Tropical Diseases, La Paz University Hospital, La Paz Institute for Health Research (IdiPAZ), Madrid, Spain; 3 Center for Biomedical Research in the Infectious Diseases Network (CIBERINFEC), Instituto de Salud Carlos III (ISCIII), Universidad Autónoma de Madrid, Madrid, Spain; 4 Small Animal Hospital, University of Glasgow, Glasgow, United Kingdom; 5 Department of Infectious Diseases and Global Health, Centro de Investigación en Sanidad Animal (CISA-INIA), CSIC, Valdeolmos, Spain; 6 Facultad HM de Ciencias de la Salud, Universidad Camilo José Cela, Madrid, Spain; 7 Instituto de Investigación Sanitaria HM Hospitales, Madrid, Spain

**Keywords:** canine pathogens, companion animals, epidemiology, One Health, pre-existing immunity, SARS-CoV-2, seroprevalence, transmission dynamics

## Abstract

**Introduction:**

This exploratory study investigated SARS-CoV-2 transmission dynamics and risk factors in companion animals from COVID-19-positive households in Madrid, Spain. The research was conducted during the early pandemic lockdown, providing a unique scenario of intensified human-animal cohabitation within domestic “transmission units.”

**Methods:**

A total of 87 pets (18 cats and 69 dogs) were enrolled through voluntary recruitment in veterinary clinics in Madrid. Exposure was assessed using RT-qPCR from nasopharyngeal swabs and the detection of specific IgM/IgG antibodies against SARS-CoV-2. Humoral immunity against common canine pathogens (CAV, CPV, and CDV) was also evaluated. Owners completed epidemiological questionnaires regarding pet habits, environment, and owner-reported clinical signs to assess potential risk factors.

**Results:**

While all animals were negative by RT-qPCR, specific antibodies were detected in 27.8% (5/18; 95% CI: 12.5–50.9%) of cats and 30.4% (21/69; 95% CI: 20.8–42.1%) of dogs. Seroprevalence was comparable between households of healthcare and non-healthcare workers (36.4% vs. 33.3% in dogs). No significant associations were found between individual risk factors and SARS-CoV-2 seropositivity. However, in dogs, reported symptoms were significantly associated (*p* < 0.05) with vaccination status, social contact, and diet. Notably, a strong and highly significant association (*p* < 0.001; V > 0.6) was observed between SARS-CoV-2 humoral immunity and antibodies against common canine pathogens (CAV, CPV, and CDV).

**Discussion:**

These exploratory findings suggest that pets are integral members of domestic transmission units, showing high exposure levels regardless of the owner’s healthcare professional status. The correlation observed with endemic canine pathogens may be associated with the pet’s immune background, although this requires further mechanistic validation through longitudinal studies. Given the limited sample size, particularly in the feline cohort, these results offer preliminary insights into the multifactorial nature of infection at the human-animal interface and support the value of integrated One Health surveillance to enhance global preparedness against zoonotic threats.

## Introduction

Severe acute respiratory syndrome coronavirus 2 (SARS-CoV-2), first identified in 2019, triggered a global pandemic with over 779 million cases as of 2024 (1). Beyond human impact, natural infections have been reported globally in a broad range of domestic, captive, and wildlife species, indicating frequent reverse zoonosis or “zooanthroponosis” (2–4). As documented by the World Organisation for Animal Health (WOAH), these infections span four continents, necessitating ongoing surveillance to monitor potential new reservoirs and virus transmission dynamics (5).

One of the world’s current major health concerns is the increase in the number of zoonotic diseases. Over the last six decades, the rate of disease emergence has considerably increased (6), and animals have played a crucial role in this phenomenon as spreaders, reservoirs, or new hosts of diseases with zoonotic potential (7). For this reason, understanding the role of animals in the dynamics of new diseases shared with humans is imperative to improve disease control and prevention. This is best achieved through a “One Health” approach, which integrates veterinary medicine, public health, and environmental health, as the three components influence the emergence and spillover of zoonotic pathogens (6). SARS-CoV-2 exemplifies the need for such a comprehensive approach as it is a novel coronavirus that has shown the ability to easily transmit from humans to more than 30 species of wild and domestic animals. Moreover, the virus has been able to spill back from animals to humans as demonstrated in farmed minks, pet hamsters or red deer (3). Understanding the various scenarios of transmission from humans to animals is crucial to preparing for potential future outbreaks.

**Figure 1:**
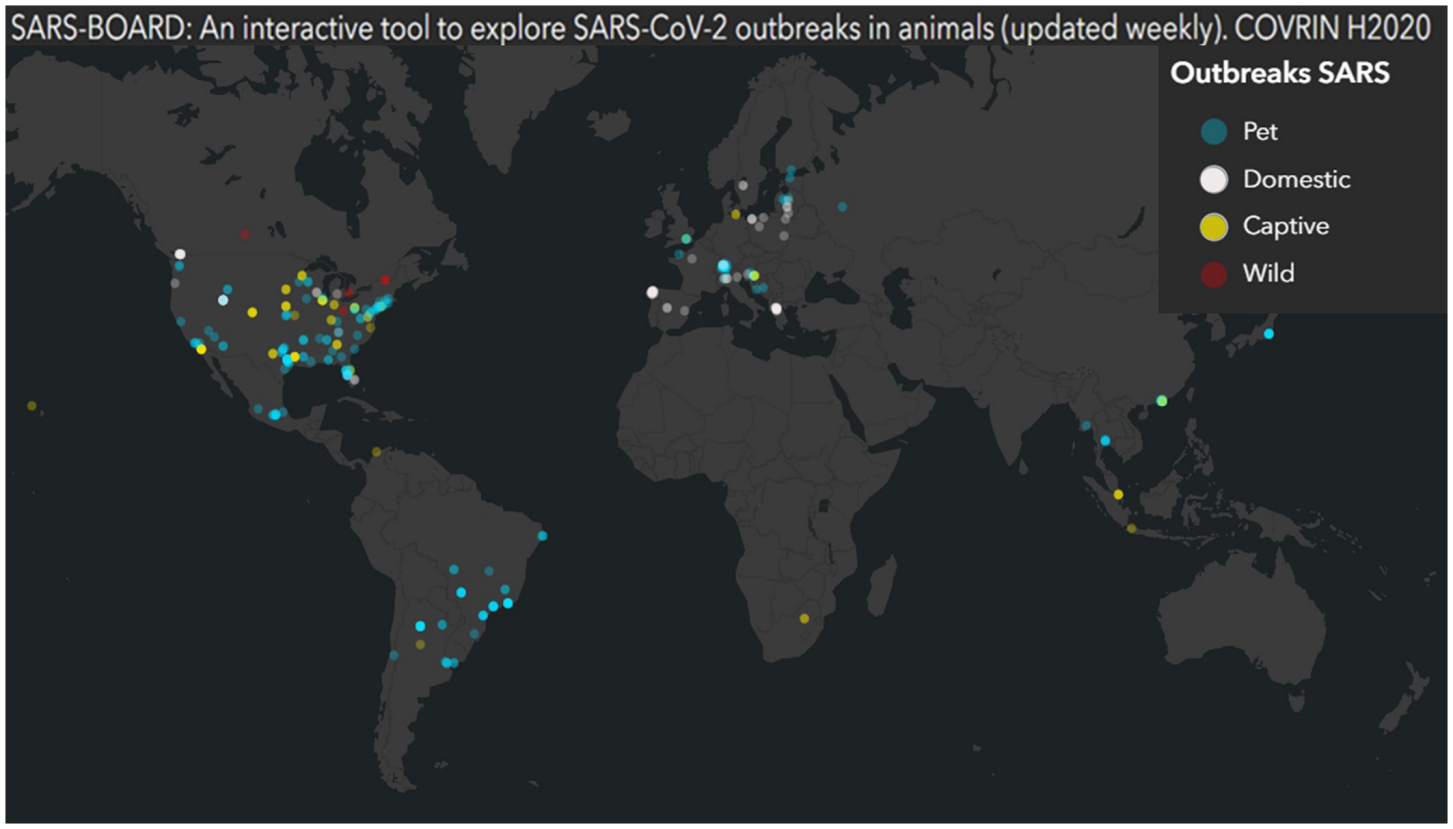
Global distribution of SARS-CoV-2 notifications in animals from January 2020 to April 2026. Data are sourced from the SARS-BOARD dashboard [CISA-INIA, CSIC; Iglesias, (35)]. Outbreaks categories are represented by colored dots: wildlife (red), captive animals (yellow), domestic livestock or fur farms (white), and pets (blue).

Domestic animals are susceptible to various species-specific coronaviruses (such as canine enteric (CECoV) and respiratory (CRCoV) coronaviruses, or feline infectious peritonitis virus (FCoV)) and their susceptibility to SARS-CoV-2 is also well-confirmed (8–13). Cats generally exhibit higher susceptibility and aerosol transmission potential, whereas dogs often display asymptomatic infections (14–20). Globally, PCR-positive cats were often in close contact with COVID-19-positive individuals. As of 2026, the WOAH has reported 506 outbreaks in 34 countries, primarily involving pets in close contact with infected individuals (72%). This data underscores the impact of SARS-CoV-2 across a wide range of animal species, particularly among dogs and cats (21) (Figure 1). Identifying risk factors within these human-pet interactions is crucial for informing public health and One Health strategies (14, 22–28).

Ongoing surveillance is essential to assess the role of domestic animals as potential reservoir hosts (5); This aspect is particularly important within “transmission units” that represent households, including pets and their owners (29). While the sample size, particularly within the feline cohort (*n* = 18), reflects the logistical challenges of recruitment during a public health crisis, this study offers a unique and exceptional scenario for investigation. In contrast to most evidence generated after mobility restrictions were relaxed, our research was conducted during the early phase of the pandemic under strict lockdown conditions in Madrid. During this period, close cohabitation was markedly intensified, providing a valuable maximal contact model that justifies the exploratory analysis of SARS-CoV-2 exposure in these pets despite the limited population size (30, 31), where mobility was severely restricted and close cohabitation between owners and pets was markedly intensified. This unique context provides a valuable opportunity to assess SARS-CoV-2 exposure in pets under conditions of maximal human–animal contact.

Therefore, this exploratory study aims to explore the epidemiology and risk factors of SARS-CoV-2 among pets living in households, the primary “transmission unit” where prolonged close contact enables viral spread. Specifically, we compared pets from healthcare and non-healthcare worker households in Madrid to evaluate potential differences in domestic exposure levels under these exceptional conditions. Furthermore, this research seeks to evaluate how the pre-existing immunological background of companion animals (specifically immunity against common canine endemic pathogens) may be associated with SARS-CoV-2 exposure, providing fresh insights into domestic infection dynamics at the human-pet interface.

## Materials and methods

### Epidemiological study

This study constitutes an exploratory epidemiological assessment conducted during the strict COVID-19 lockdown in Madrid, Spain. Dogs and cats co-living with at least one SARS-CoV-2 infected owner (COVID-19-positive households) were enrolled through voluntary recruitment via a network of veterinary clinics and hospitals in Madrid. These animals were considered SARS-CoV-2 exposed animals, as they cohabited within the household, defined as the primary domestic “transmission unit,” where close and prolonged contact with infected owners occurred. While acknowledging potential selection bias inherent in voluntary participation, this strategy provided a unique opportunity to evaluate pets as SARS-CoV-2 exposed individuals.

Blood and nasopharyngeal swabs from exposed pets were collected between June 2020, and March 2021. Samples from dogs not exposed to SARS-CoV-2 were collected before September 2019 for another veterinary study and were used under the owners’ renewed permission. Once the pet was sampled, the owners completed a questionnaire with basic data about the pet, health information and lifestyle habits to evaluate possible risk factors associated with SARS-CoV-2 infection of companion animals. The owners’ infection was confirmed by a rapid antigen test or by RT-qPCR. Some of SARS-CoV-2-positive owners were healthcare workers at the Hospital Universitario La Paz in Madrid (Spain). Pet sampling was typically performed between 1 and 3 weeks after the initial human diagnosis, as quarantine restrictions and logistics (such as the absence of non-quarantined cohabitants to transport the animals) often delayed access to the pets during the acute phase of household infection.

The study protocol was approved in June 2020 by the Ethics Committee of the Faculty of Health Sciences, Universidad Alfonso X el Sabio. All owners provided written informed consent for the collection and processing of pet samples, as well as for the use of epidemiological data, ensuring compliance with ethical standards for animal research.

### RT-qPCR analysis

Nasopharyngeal swabs from SARS-CoV-2 exposed pets (*n* = 61) were collected and analyzed by RT-qPCR at Laboklin or at CISA-INIA facilities to determine the presence of SARS-CoV-2. In swabs analyzed at Laboklin, RNA and DNA were automatically isolated using the MagNA Pure 96 system (Roche Diagnostics). The presence of SARS-CoV-2 was tested by Taqman real-time PCR on a LightCycler^®^96 (Roche Diagnostics). RNA from samples analyzed at CISA-INIA were co-extracted with an exogenous internal positive control (IPC-EGFP) using IndiSpin Pathogen Kit (Indical Bioscience). Then, viral RNAs were analyzed by an *in-house* triplex real-time RT-PCR (based on Centers for Disease Control and Prevention 2019-nCoV: https://www.fda.gov/media/134922/download) for the simultaneous detection of SARS-CoV-2 (N1 gene), an internal control (beta-actin gene), and the exogenous control (EGFP). For both PCR-based methods, samples with Ct value >40 were considered negative.

### Serological assays

Detection of IgM and IgG antibodies against SARS-CoV-2, and IgG antibodies against common canine endemic pathogens, as canine adenovirus (CAV), canine parvovirus (CPV) and canine distemper virus (CDV) were analyzed following the protocol previously described (32). Antibody levels were classified as negative (<0), mild positive (1–20), positive (21–73) and high positive (74–100).

### Epidemiological questionnaire

Owners completed standardized questionnaire covering three areas: (1) general pet information (species, breed, sex, age, and weight); (2) environment and habits (walking, resting, and feeding patterns, and social contact); and (3) clinical health data, including respiratory and digestive symptoms, behavioral changes, and vaccination status. Symptoms from the questionnaire were documented as owner-reported clinical signs.

### Statistical analysis

Variables were categorized according to Supplementary Table S1. Descriptive analysis for both cats and dogs utilized absolute and relative frequencies. The normal distribution of age was assessed with the Shapiro–Wilk test, and categorized age groups were used for the analyses. Associations between categorical variables (seropositivity, symptoms and risk factors) were evaluated using chi-square test and Cramer’s V coefficient: no association (0–0.2), weak association (0.2), moderate association (0.2–0.6) and strong association (0.6–1). This coefficient was specifically selected because it provides a robust measure of the strength of association in contingency tables and small sample sizes. This statistical choice ensures that the inferences drawn from the feline cohort (*n* = 18) and canine population were statistically appropriate. All tests were considered statistically significant at *p* ≤ 0.05 (95% significance level). Analyses and graphical representations were performed using Stata^®^16.1 (StataCorpLLC, United States) and GraphPad Prism8.0.1.

## Results

### Study cat population

**Figure 2:**
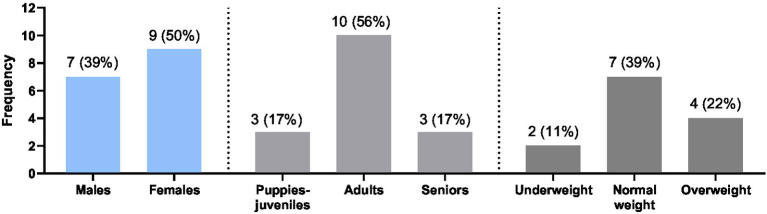
Demographic characteristics of the study feline population (*n* = 18). Frequency distribution (%) of the cohort according to sex, age category, and body condition. “Do not Know/No Answer” (DK/NA) categories are excluded from the percentage visualization.

Eighteen cats from COVID-19-positive households were enrolled. Detailed demographic characteristics, including sex, age, body condition, and breed, are summarized in Supplementary Table S2. Briefly, the cohort included of 7 males and 9 females; 3 were puppies-juveniles, 10 were adults and 3 were seniors (Supplementary Table S2; Figure 2). There was no information from two animals. Regarding body condition, 2 cats were underweighted, 7 were normal weight while 4 were overweight (Supplementary Table S2; Figure 2). Most cats were Common European breed (50%), although a Siamese, a Persian, a British Shorthair and a mixed breed were also included (each breed represented 5.55%) (Supplementary Table S2).

Regarding the cats’ environment and living habits, notably, 44.44% lived with healthcare workers. The majority remained indoors (94.4%) and co-slept with their owners (61.1%). Social interaction was common (72.22% with other people; 33.33% with animals), and diet was predominantly commercial (55.6%).

### Study canine population

**Figure 3:**
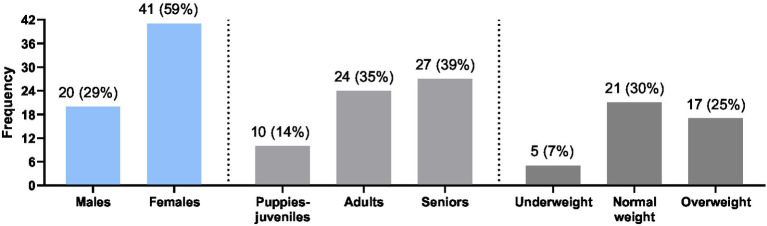
Demographic characteristics of the study canine population (*n* = 69). Frequency distribution (%) of the cohort according to sex, age category, and body condition. “Do not Know/No Answer” (DK/NA) categories are excluded from the percentage visualization.

Sixty-nine dogs from SARS-CoV-2-infected owners were enrolled. Detailed demographic characteristics, including sex, age, body condition, and breed, are summarized in Supplementary Table S3. There was no information of 7 animals and was not complete for other 19. Briefly, the population was primarily composed of females (≥59.4%) and seniors (≥39.1%) (Figure 3). Most companion dogs were crossbreeds (30.43%), followed by Dachshund (5.79%), Beagle (2.89%), Golden Retriever (2.89%), and German Shepherd (2.89%) (Supplementary Table S3).

Regarding canine environmental and lifestyle characteristics, nearly half of the owners (47.8%) were healthcare professionals. Most dogs walked outside daily (79.7%), maintained frequent social contact (81.2% with people; 68.1% with animals), and slept independently (58%). Diet was primarily commercial (55.1%).

### Vaccination, clinical signs, and SARS-CoV-2 immunity

**Figure 4:**
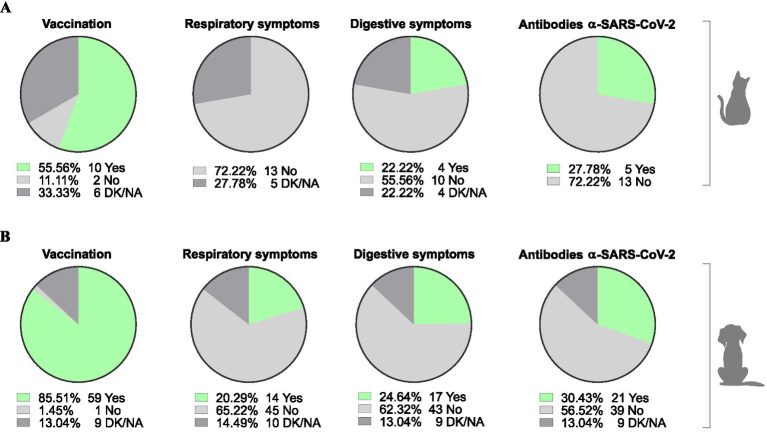
Immunological status, symptomatology, and SARS-CoV-2 serological results. Percentage distribution of vaccination status, owner-reported clinical signs (respiratory and digestive), and SARS-CoV-2 specific antibody detection in cats **(A)** and dogs **(B)**. DK/NA: Do not Know/No Answer.

Regarding health and welfare cat information provided by the owners, most cats were vaccinated (55.56%) and respiratory-asymptomatic, although 22.22% showed gastrointestinal signs (Figure 4A). While all nasopharyngeal swabs (*n* = 15) were RT-qPCR negative, specific antibodies were detected in 27.78% (5/18; 95% CI: 12.5–50.9%) of the feline population (Figure 4A). Notably, the seroprevalence in healthcare-worker households was 25.0% (2/8).

Concerning the canine health status, most dogs (85.5%) were vaccinated and showed protective immunity against common pathogens (CAV, CPV, CDV) (Figure 4B). Respiratory and digestive symptoms were reported in 20.3 and 24.6% of animals, respectively. While all oropharyngeal swabs (*n* = 46) were RT-qPCR negative, SARS-CoV-2 antibodies were detected in 30.4% (21/69; 95% CI: 20.8–42.1%) of the population, including 28 cases previously analyzed (32) (Figure 4B). Seroprevalence was comparable between healthcare (36.4%) and non-healthcare (33.3%) households.

### Variable associations

**Figure 5:**
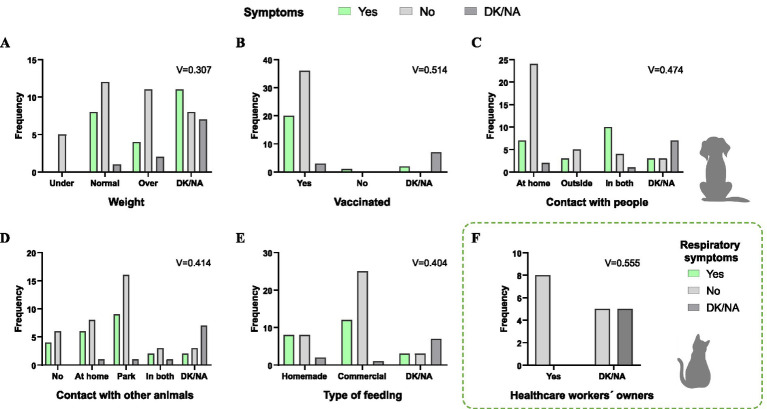
Bivariate associations between pet symptomatology and epidemiological variables. Frequency of clinical signs in dogs according to body condition **(A)**, vaccination status **(B)**, contact with other people **(C)**, contact with other animals **(D)**, and type of feeding **(E)**. Association between respiratory symptoms in cats and owners’ healthcare profession **(F)**. Statistical strength was assessed using Cramer’s V (values between 0.2 and 0.6 indicate a moderate association). DK/NA: Do not Know/No Answer.

No association was found between the presence of antibodies against SARS-CoV-2 (humoral immunity) and any of the variables analyzed, neither in cats nor in dogs. Regarding canine symptoms, body weight showed a moderate but non-significant association (*p* = 0.053; V = 0.307) (Figure 5A). In contrast, preliminary associations (*p* < 0.05) were identified with vaccination status, contact with people and other animals, and type of food (Figures 5B–E). According to Cramer’s V, these associations were all moderate in strength (V = 0.404–0.514). In cats, a significant (*p* = 0.036) and moderate association (V = 0.555) was found between the presence of respiratory symptoms and the healthcare profession of their owners (Figure 5F).

**Figure 6:**
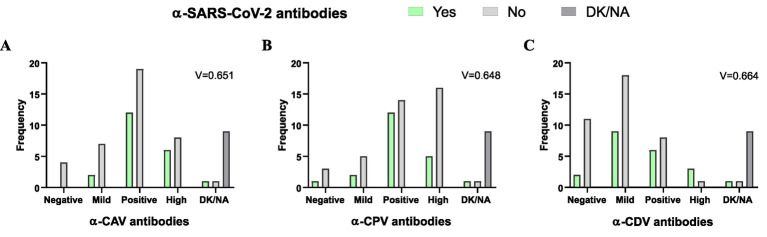
Correlational analysis between SARS-CoV-2 antibodies and endemic canine pathogens. Association between the presence of *α*-SARS-CoV-2 antibodies and antibodies levels for CAV **(A)**, CPV **(B)**, and CDV **(C)**. DK/NA: Do not know/No answer. Cramer’s V > 0.6 indicates a strong association.

Notably, strong and highly significant associations (*p* < 0.001) were found between SARS-CoV-2 antibodies and antibodies against CAV, CPV, and CDV (Figures 6A–C). According to Cramer’s V, these associations were all strong in strength, with values ranging from 0.648 to 0.664.

## Discussion

This study provides exploratory insights into SARS-CoV-2 transmission at the human-pet interface within domestic environments. Given its nature as a hypothesis-generating assessment, these findings offer a foundational understanding of how domestic “transmission units” functioned during a period of unique and intensified cohabitation. While the limited feline cohort size (*n* = 18) reduces the statistical power to detect subtle associations, our data provide valuable evidence of SARS-CoV-2 exposure in companion animals during the early pandemic phase in Madrid. Key observations include a relatively high seroprevalence in both dogs and cats, no significant differences in exposure between healthcare and non-healthcare worker households, and the absence of detectable viral RNA at the time of sampling despite evidence of past humoral response.

Since the onset of the COVID-19 pandemic, the susceptibility of various animal species to natural SARS-CoV-2 infection has been well-established; as of 2024, the World Organization for Animal Health (WOAH) has documented 447 outbreaks across 34 countries, involving a wide range of domestic and wild species. Immune responses to the virus have been reported in several domestic cats and dogs worldwide, with close contact with COVID-19-infected humans as the main route of infection (23, 28, 33, 34). In this context, surveillance data from European One Health initiatives suggest that SARS-CoV-2 transmission from infected owners to pets can occur, although it is generally uncommon and linked to prolonged close contact within households (35). Understanding the transmission pathways from humans to animals and the risk factors associated with viral infection in domestic animals is crucial, not only to ensure animal health, but also to prevent viral adaptation in pets and the potential establishment of animal reservoirs.

Our study detected specific antibodies in 27.78% (5/18) of cats and 30.43% (21/69) of dogs, confirming that pets in close contact with infected humans can develop specific immune responses (36, 37). Cats have generally been considered more susceptible to SARS-CoV-2 infection than dogs, supported by receptor-binding predictions and experimental evidence (38, 39). While some reports suggest higher seroprevalence in cats, the broad circulation within these “transmission units” underscores the need for multi-species surveillance (40). The seroprevalence observed in this study is slightly higher in dogs than in cats. Seroprevalence studies conducted on cats and dogs worldwide have reported varying positivity rates, which are generally lower in both species compared to those observed in our study. Specifically, feline seroprevalence (~28%) exceeded rates in Italy (4.5%), Portugal (10.1%), and Wuhan (20%), but was lower than in Texas (43.8%) (37, 41–43). Similarly, canine rates (30.4%) surpassed those found in Italy (12.8%), Portugal (2.7%), Wuhan (11.1%), and Texas (25.6%) (37, 41–43). These variations likely reflect differences in regional human incidence, sampling criteria, or diagnostic methods.

Despite the high seroprevalence observed (approximately 28% in cats and 30% in dogs) all nasopharyngeal swabs remained negative by RT-qPCR. This disparity indicates that active viral shedding was likely cleared prior to sampling or that viral loads had fallen below analytical detection thresholds. These findings highlight the transient nature of active infection at the human–pet interface and emphasize the importance of using serological markers to accurately assess exposure within domestic transmission units.

While previous reports have suggested that SARS-CoV-2 infection is more prevalent in adult and male dogs (37), our results did not identify a significant association between host age or sex and the development of a specific humoral immune response. This absence of association is consistent with the finding reported by Barroso et al. (41). Moreover, our study detected similar rates of humoral immunity against SARS-CoV-2 in dogs from households with healthcare professionals and those from households where owners work in other fields (36% *versus* 33%). This result suggests that the apparent higher exposure to the virus of owners in sanitary environment may not play such an important role on canine immunity as might be hypothesized (44, 45).

Previous studies linked SARS-CoV-2 seropositivity to the duration and closeness of human-animal contact. In Ontario, pet seropositivity correlated with clinical signs (respiratory, gastrointestinal, or lethargy) (46). While co-sleeping was a risk factor specifically for cats, no associations were found regarding multi-pet households, number of infected owners, or daily contact duration (46). Other studies associated infection with owner cohabitation, outdoor activities, or social contact (23, 26). Despite these insights, varying results across studies prevent from drawing definitive conclusions about the risk factors for pet infection (41).

SARS-CoV-2-infected pets are typically asymptomatic or mildly symptomatic (33, 43, 47, 48), though severe respiratory or digestive symptoms can develop, primarily in cats (32, 41). This study identified moderate associations in dogs between symptoms and factors like weight, social contact and vaccination, plus a strong association between SARS-CoV-2 immunity and endemic canine pathogens, findings not previously highlighted (46). Notably, the strong and highly significant association observed between SARS-CoV-2 humoral immunity and antibodies against CAV, CPV, and CDV (V > 0.6) warrants a deeper mechanistic interpretation. Rather than direct viral cross-reactivity, this correlation likely reflects the overall immunological fitness or immune background of the animals. High titers against endemic pathogens may serve as a proxy for comprehensive vaccination compliance and frequent veterinary care, which in turn could be associated with owners who are more proactive in monitoring their pets’ health during the pandemic. Future studies should investigate whether a primed immune system through routine vaccination provides a non-specific heterologous advantage or if it simply identifies a subpopulation of pets with better-maintained health status and potentially different environmental exposure patterns. Similarly, the link between feline respiratory signs and healthcare-worker households is novel and suggests increased viral exposure (26, 45). These results offer preliminary insights into potential correlations between environmental factors, pre-existing immunity, and domestic infection dynamics.

Several limitations of this study warrant consideration. First, while the limited sample size, particularly in the feline cohort, may have reduced the statistical power to detect subtle associations, this is balanced by the uniqueness of the sampling period. Our research captured a maximal contact domestic scenario during the early 2020 lockdown in Madrid that is no longer reproducible, providing a valuable model for pure household transmission. In addition, although multivariable analyses were considered, the limited number of seropositive animals (particularly in cats), together with incomplete responses and multiple-category epidemiological variables, resulted in an insufficient number of outcome events for robust multivariable modeling. Under these conditions, multivariable analyses would have been statistically underpowered and at risk of overfitting; therefore, only exploratory bivariate analyses were performed. Second, the timing of sample collection likely influenced the diagnostic outcomes. Due to mandatory quarantine and the lack of available cohabitants to transport pets to veterinary clinics, most animals were sampled between 1 and 3 weeks after the owners’ diagnosis. This timeframe explains why active viral shedding was no longer detectable by RT-qPCR in nasopharyngeal and oropharyngeal swabs, while specific IgM and IgG antibodies were already well-established at the time of sampling. This suggests that active viral shedding may have ceased or dropped below detection thresholds prior to sampling. Third, the reliance on owner-reported clinical signs introduces subjectivity; yet, under strict lockdown conditions, this was the only viable method to gather real-time observational data on pet health. Despite these constraints, as an exploratory and hypothesis-generating assessment, this study offers a critical foundation for understanding viral dynamics at the human-pet interface.

Although active viral shedding was not detected via RT-qPCR, the high seroprevalence observed (approximately 30% in both dogs and cats) confirms that pets are integral participants in domestic transmission units and susceptible hosts for reverse zoonosis. The fact that exposure levels remained consistent regardless of the owners’ professional healthcare status, suggests that close household contact is the primary driver of transmission. Furthermore, the strong associations found between SARS-CoV-2 immunity and other endemic canine pathogens (CAV, CPV, and CDV) suggest that host-related or environmental factors (such as immune background, vaccination status, or shared exposure conditions) may be associated with domestic infection dynamics, highlighting the multifactorial and correlational nature of pathogen circulation within the human-pet interface. From a 2026 perspective, our data support the One Health imperative for integrated surveillance. The consistency of seroprevalence across different owner professions suggests that the virus behaves as a highly efficient domestic unit traveler, regardless of specific human professional exposure levels. This underscores that veterinary practitioners must be central to public health strategies to monitor the potential role of companion animals in viral circulation and enhance global preparedness against future zoonotic threats.

## Conclusion

In conclusion, this exploratory study provides preliminary evidence of high viral exposure within domestic transmission units, where approximately 30% of pets in infected households developed specific antibodies. While the limited sample size, particularly in the feline cohort, warrant a cautious interpretation, the finding that seroprevalence remained consistent regardless of the owners’ healthcare profession suggests that close household contact is the primary driver of transmission. Furthermore, the observed associations between SARS-CoV-2 immunity and other common canine pathogens offer potential insights for future integrated surveillance. These results support the value of a One Health approach in multi-species monitoring to enhance global preparedness for future zoonotic outbreaks.

## Data Availability

The original contributions presented in the study are included in the article/Supplementary material, further inquiries can be directed to the corresponding author.
