# Supplementary material for: SARS-CoV-2 at the human-pet interface: transmission and pre-existing immunity in pets within infected households
Source: Front Vet Sci. 2026 Jul 20;13:1862845. doi: 10.3389/fvets.2026.1862845 (PMC13430146; doi:10.3389/fvets.2026.1862845)
Supplement: Supplementary file 1 [file Supplementary_file_1.zip › Supplementary Material S1/Supplementary Tables 1-3.docx]

**Table S1. Detailed description of variables and categorizations included in the statistical analysis.** DK/NA: Don´t Know/No Answer.

| **Nº** | **Variable** | **Categorization** |
| --- | --- | --- |
| **1** | Age (years) | N/A |
| **2** | Age categorized for dogs | 0: Puppy-juvenile: 0 to 2 years old.  1: Adult: 2.1 to 7 years old  2: Senior: more than 7.1 years old  3: DK/NA |
| **3** | Age categorized for cats | 0: Puppy-juvenile: 0 to 2 years old.  1: Adult: 2.1 to 10 years old  2: Senior: more than 10.1 years old  3: DK/NA |
| **4** | Pet owner by profession healthcare worker | 0: No  1: Yes  2: DK/NA |
| **5** | Species of animal | 0: Dog  1: Cat |
| **6** | Sex of animal | 0: Male  1: Female  2: DK/NA |
| **7** | Body weight condition | 0: Underweight  1: Normal weight  2: Overweight  3: DK/NA |
| **8** | Antibodies against SARS-CoV-2 | 0: Negative  1: Positive  2: DK/NA |
| **9** | Antibodies against CAV | 0: Negative  1: Mild positive  2: Positive  3: High positive  4: DK/NA |
| **10** | Antibodies against CPV | 0: Negative  1: Mild positive  2: Positive  3: High positive  4: DK/NA |
| **11** | Antibodies against CDV | 0: Negative  1: Mild positive  2: Positive  3: High positive  4: DK/NA |
| **12** | Evidence of symptoms | 0: No  1: Yes  2: DK/NA |
| **13** | Respiratory symptoms | 0: No  1: Yes  2: DK/NA |
| **14** | Digestive symptoms | 0: No  1: Yes  2: DK/NA |
| **15** | Vaccination status | 0: No  1: Yes  2: DK/NA |
| **16** | Walking habit | 0: Does not go outside  1: Out daily  2: DK/NA |
| **17** | Walking places | 0: Country  1: City  2: Country and city  3: DK/NA |
| **18** | Place where animal usually stay | 0: Outside home  1: Home  2: Both  3: DK/NA |
| **19** | Place where animal usually sleeps | 0: Outside home  1: Home  2: DK/NA |
| **20** | Place where animal usually sleeps inside the house | 0: Co-sleeping with owner  1: Sleeping independently  2: DK/NA |
| **21** | Type of animal feeding | 0: Includes homemade food  1: Commercial food only  2: DK/NA |
| **22** | Type of commercial food | 0: Dry  1: Wet  2: Both  3: DK/NA |
| **23** | Usual contact with other animals | 0: No  1: Yes, at home  2: Yes, in the park  3: Yes, at home; yes, in the park  4: DK/NA |
| **24** | Usual contact with other people | 0: No  1: Yes, at home  2: Yes, outside  3: Yes, at home; yes, outside  4: DK/NA |

**Table S2. Individual characteristics and demographic data of the participant feline cohort (n=18).** DK/NA: Don´t Know/No Answer.

| **Cat** | **Sex** | **Age group** | **Body condition** | **Breed** |
| --- | --- | --- | --- | --- |
| **K108** | M | Senior | Normal weight | Common European |
| **K224** | DK/NA | DK/NA | DK/NA | DK/NA |
| **K225** | M | Senior | Normal weight | Mixed breed |
| **K43** | M | Adult | Normal weight | Common European |
| **K46** | M | Adult | Overweight | Common European |
| **K48** | F | Adult | Overweight | Common European |
| **K58** | F | Puppy-juvenile | Normal weight | Common European |
| **K59** | F | Adult | Underweight | Common European |
| **K69** | F | Adult | Normal weight | Persian |
| **K70** | F | Puppy-juvenile | Normal weight | Siamese |
| **K81** | F | Adult | DK/NA | DK/NA |
| **K82** | F | Senior | DK/NA | DK/NA |
| **K83** | F | Adult | DK/NA | DK/NA |
| **K89** | M | Adult | Overweight | Common European |
| **K93** | DK/NA | DK/NA | DK/NA | DK/NA |
| **K94** | M | Adult | Overweight | Common European |
| **K95** | F | Adult | Underweight | Common European |
| **K96** | M | Puppy-juvenile | Normal weight | British Shorthair |

**Table S3. Individual characteristics and demographic data of the participant canine cohort (n=69).** DK/NA: Don´t Know/No Answer.

| **Dog** | **Sex** | **Age group** | **Body condition** | **Breed** |
| --- | --- | --- | --- | --- |
| **68** | F | Adult | Overweight | Mixed breed |
| **71** | M | Senior | Overweight | Ratonero Bodeguero Andaluz |
| **73** | M | Puppy-juvenile | Normal weight | Mixed breed |
| **K226** | M | Senior | DK/NA | Mixed breed |
| **K227** | F | Adult | Underweight | Greyhound |
| **K41** | F | Puppy-juvenile | Normal weight | Spanish Water Dog |
| **K42** | F | Senior | DK/NA | Mixed breed |
| **K44** | F | Senior | Normal weight | Pug |
| **K47** | F | Adult | DK/NA | Mixed breed |
| **K49** | F | Senior | Normal weight | Labrador Retriever |
| **K50** | F | Adult | Underweight | Mixed breed |
| **K51** | F | Adult | Overweight | Yorkshire Terrier |
| **K52** | F | Senior | Overweight | Bichon Maltese |
| **K53** | F | Adult | Normal weight | Podenco |
| **K55** | F | Senior | DK/NA | Mixed breed |
| **K56** | F | Adult | Overweight | Labrador Retriever |
| **K57** | M | Senior | Overweight | Dachshund |
| **K64** | M | Adult | Normal weight | Toy Poodle |
| **K65** | M | Senior | Underweight | Schnauzer |
| **K66** | M | Adult | Normal weight | Dachshund |
| **K67** | M | Adult | Overweight | Mixed breed |
| **K68** | F | Puppy-juvenile | Overweight | Shih Tzu |
| **K71** | DK/NA | DK/NA | DK/NA | DK/NA |
| **K72** | DK/NA | DK/NA | DK/NA | DK/NA |
| **K73** | DK/NA | DK/NA | DK/NA | DK/NA |
| **K74** | DK/NA | DK/NA | DK/NA | DK/NA |
| **K75** | F | Puppy-juvenile | Overweight | Ratonero Bodeguero Andaluz |
| **K76** | M | Adult | Normal weight | Pug |
| **K77** | DK/NA | DK/NA | DK/NA | DK/NA |
| **K78** | F | Adult | Overweight | Yorkshire Terrier |
| **K79** | M | Adult | Normal weight | Mastiff |
| **K80** | M | Senior | DK/NA | Mixed breed |
| **K84** | F | Senior | Normal weight | Bull Terrier |
| **K85** | DK/NA | DK/NA | DK/NA | DK/NA |
| **K86** | DK/NA | DK/NA | DK/NA | DK/NA |
| **K87** | F | Adult | Normal weight | Border Collie |
| **K88** | F | Senior | DK/NA | Mixed breed |
| **K90** | F | Senior | Normal weight | Weimaraner |
| **K91** | F | Senior | DK/NA | Mixed breed |
| **K92** | F | Senior | DK/NA | Mixed breed |
| **K97** | M | Senior | DK/NA | Mixed breed |
| **SER101** | F | Senior | Underweight | Podenco |
| **SER102** | M | Senior | DK/NA | Mixed breed |
| **SER103** | F | Adult | Normal weight | Golden Retriever |
| **SER104** | F | Puppy-juvenile | Underweight | Labrador mixed |
| **SER105** | F | Adult | Normal weight | Jack Russell Terrier |
| **SER106** | M | Senior | Normal weight | Beagle |
| **SER107** | F | Adult | Normal weight | German Shepherd |
| **SER108** | F | Senior | Overweight | French Bulldog |
| **SER109** | F | Adult | Overweight | Pekingese |
| **SER110** | M | Puppy-juvenile | Normal weight | Dachshund mixed |
| **SER111** | F | Senior | DK/NA | Shih Tzu |
| **SER112** | F | Senior | DK/NA | Boxer |
| **SER113** | F | Adult | Normal weight | Border Collie |
| **SER114** | M | Puppy-juvenile | Normal weight | Golden Retriever |
| **SER115** | F | Adult | Overweight | Bichon Maltese |
| **SER116** | F | Adult | DK/NA | Mixed breed |
| **SER117** | M | Adult | Overweight | Spanish Water Dog |
| **SER118** | F | Puppy-juvenile | DK/NA | Mixed breed |
| **SER119** | F | Adult | DK/NA | Mixed breed |
| **SER120** | F | Adult | Overweight | Mixed breed |
| **SER121** | F | Puppy-juvenile | Overweight | Fox Terrier |
| **SER126** | M | Senior | Normal weight | Pit Bull |
| **SER127** | M | Senior | DK/NA | Dachshund |
| **SER128** | F | Senior | DK/NA | Dachshund |
| **SER129** | F | Puppy-juvenile | DK/NA | Mastiff mixed |
| **SER130** | F | Senior | Overweight | German Shepherd |
| **SER14** | DK/NA | DK/NA | DK/NA | English Cocker Spaniel |
| **SER21** | M | Senior | Normal weight | Beagle |
